# A Scoping Review: Do instruments measuring genomic competence in nursing incorporate ethics?

**DOI:** 10.1002/nop2.1805

**Published:** 2023-05-23

**Authors:** Mari Laaksonen, Elisa Airikkala, Arja Halkoaho, Eija Paavilainen

**Affiliations:** ^1^ Faculty of Social Sciences, Health Sciences Unit, Etelä‐Pohjanmaa Hospital district Tampere University Tampere Finland; ^2^ Tampere University of Applied Sciences Tampere Finland

**Keywords:** ethical competence, genomic competence, measure, nursing, scoping review

## Abstract

**Aim:**

To explore the instruments used in measuring genomic competence in nursing. The objective was to understand how ethical issues are reflected in the instruments.

**Design:**

A scoping review.

**Methods:**

A systematically conducted scoping review was conducted by using CINAHL Complete and Medline databases covering the years from January 2010 to January 2022. Two authors inspected potentially eligible papers and assessed their quality independently using the critical appraisal tools of Joanna Briggs Institute. Twenty‐five articles were eligible including 19 different instruments. Included articles answered the research question: “How ethical issues are reflected in instruments measuring genomic competence in nursing?” The inductive thematic analysis was used in this review.

**Results:**

Descriptions of ethical themes were unstructured in the scoped articles and instruments. Not all genomic competence instruments covered ethical aspects. Only three studies asked direct questions by using the word ethics or its derivates, including confidentiality in solving ethical problems, familiarity with the ethical aspects of genetic counselling and ability to identify ethical issues. Thirteen articles included ethics‐related themes encompassing knowledge, skills, concerns, advantages and disadvantages.

## 1 INTRODUCTION

The study of genomics, that is, the study of the entire human genome, has developed rapidly, and the obtained data are increasingly used in health promotion, prevention and treatment of diseases (World Health Organization [WHO], 2022). As nurses work daily with patients, they play a vital role in implementation of genome‐based information and achievement of the goals of genomics to improve health outcomes of the patients (Calzone, Kirk, et al., 2018a; Whitley et al., 2020). To accomplish these goals, nurses need to acquire new competences. Nurses are required to have skills in collecting information about families' medical history, identifying individuals at risk for diseases and genomic factors underlying drug reactions, helping people to understand informed consent, interpretating genomic test results and carrying out individual interventions using genomic data (Calzone et al., 2010). These skills with acquired knowledge combine competence for genomics in nursing. Genomics includes sensitive issues nurses encounter at work with patients. Therefore, ethical aspects of genomics are significant to nurses' genomic competence.

In addition, it is known that there can be a notable distance between a scientific theory and practice. To narrow this gap and to effectively implement new information, like genomics, healthcare professionals must understand usefulness and effectiveness of genomics. This enables professionals to perceive these practices as worthwhile and relevant to their role (Mortell, 2018) and influences their ability to directly apply genomics in practice (Calzone et al., 2014).

Although it is vital that health professionals understand the significance of genomics as a scientific field (Consensus Panel, 2009), the majority of the healthcare providers do not have an educational background in genomics (Calzone et al., 2014; Saleh et al., 2019). In this study, the concept of genomics is used to describe both genomics and genetics unless it is necessary to make a distinction between the two.

## 2 BACKGROUND

The first guidelines for genomics competences and related suggestions for curricula for nurses were published back in the early 2000s in Europe and the United States (Consensus Panel, 2009; Kirk et al., 2003; Skirton et al., 2010). Nevertheless, the competences have not yet been fully integrated into nursing education (Camak, 2016; Kirk et al., 2011), which also means that genomics has not yet been fully integrated in nursing practice. Studies conducted in the 2010s have found that levels of genomic literacy and genomic competence in nursing are low (Calzone et al., 2012; Godino et al., 2013a; Skirton et al., 2012; Wright et al., 2019). Thompson and Brooks (2011) found that only a small share of nurses is confident in counselling clients or referring them to specialized services based on genomic data obtained from direct‐to‐consumer tests. The most recent literature shows that the gaps in integrating the genomics in nursing still remains (Calzone, Kirk, et al., 2018a; Camak, 2016; Dumo et al., 2020).

Knowledge in genomics, that is, genomic literacy, can be defined as an understanding of what the genome is and how genomic science works. This means understanding of its benefits and limitations and potential applications in health care, and the effects of genomics on society level (Ha et al., 2018; Hurle et al., 2013). Literacy can also be amplified as knowledge that includes both genomic health literacy and genomic science literacy (Hurle et al., 2013). This makes literacy a precursor to competence (Calzone, Kirk, et al., 2018b). Competence connects knowledge and applications, or knowledge and skills, into a measurable or observable entity. (Consensus Panel, 2009; Skirton et al., 2012).

Ethics can be considered a relevant part of genomic competence in at least three ways. First, the theory‐practice gap is linked with ethics in a way that Mortell (2018) calls it the theory‐practice‐ethics gap. The theory‐practice gap creates ethical dilemmas, and therefore, ethics should be considered as one component of it. As the gap may constitute a barrier for combining knowledge and skills into competence, the implementation of genomic theory into clinical practice must include ethical consideration. Nurses need to understand the competences required for practice including knowledge of ethical, legal and social issues related to genomics (Rogers et al., 2017). This enables closing the gap.

Second, ethics plays a major role in the core competences of nursing. The ethical principles of nursing include autonomy, beneficence, nonmaleficence, veracity, justice and fidelity (Cannon & Delahoyde, 2020). The internal and external factors changing the nursing profession also create constant changes in ethics (Kangasniemi et al., 2015). Changes in science and technology also create new dimensions to ethics (Cipriano, 2015). Therefore, as genomics is changing health care, ethics in health care is also changing.

Third reason is genomics itself. The genomic‐related ethical questions are already part of current practice in health care (Murakami et al., 2020). Genomics has raised new and complex ethical issues for which basic ethical concepts do not provide an answer (Huddleston, 2014; Steck, 2018). Steck (2018) raises the possibility of misinterpretation and the complexity of genomic information as examples of the complicated nature of ethical issues. Similarly, problems concerning the unauthorized dissemination of genetic information and the lack of protection of privacy have become concerns (Houwink et al., 2011). The security and confidentiality of information, health equality and the effects of genomic information on an individual have also been highlighted. Genomic data are not only about the individual but also involves considering what the information may mean to relatives, whether they have the right to receive or refuse this information, and whether there are resources in the healthcare system for meeting families or clients to discuss their newly discovered needs (Lea, 2008). Increasing knowledge of the moral and ethical implications of genomics is essential in nursing (Seven et al., 2017). In their study, Seven et al. (2017) found that 94% of nurses are still unaware of ethical regulations or a lack thereof.

Although genetic research offers undeniable benefits to consumers and the general public, in‐depth discussion is needed due to the nature of genetic information. For example, issues concerning predictive tests, interpretation of test results and how these are offered to the public raise ethical questions (Suchetana, 2021) that nurses encounter at the front line of health care. In addition, the published recommendations for genomic competences for nurses include ethics as part of identifying ethical, ethnic, cultural, religious, legal, fiscal and social issues related to genomics (Consensus Panel, 2009; Greco & Salveson, 2009).

Competences include the minimal standards for providing safe, accountable and responsible health care within a medical specialty (Calzone et al., 2012). Genomic competences require extensive skill management, which makes it difficult to integrate genomic competences, considered complex, into nursing practice (Calzone et al., 2014.) According to Wright (2015), technical, interpersonal and critical thinking are domains of skills necessary in nursing. Competence assessments should also address these skills. Therefore, there is a need to study how genomic competence is assessed in nursing. Despite many previous studies, it is unclear what kind of competence the used survey instruments measure and whether the instruments cover all components of genomic competence. Anderson et al. (2015) studied psychometrically robust survey instruments of genomic competence in their systematic review, and Skirton et al. (2012) and Wright et al. (2018) focused on competence levels in their reviews. For these reasons, this scoping review aims to identify and chart the available evidence of the content of the instruments from a new angle: the ethical competence in genomic‐informed nursing. Skirton et al. (2012) demonstrated that only a few studies, 3 out of 11, mentioned ethical aspects: discrimination of ethnic groups, ethical concerns due to the personal religious beliefs of health personnel, and a lack of an ethical protocol.

According to Munn et al. (2018), the scoping reviews reveal how studies are conducted by describing and analysing gaps in a certain area of literature. This scoping review aims to clarify the key concept of ethical competence in genomics, identify related key characteristics and describe how the key concept is understood. The research question is: How ethical issues are reflected in instruments measuring genomic competence in nursing?

## 3 THE STUDY

### 3.1 Design

The study design was a scoping review. The authors followed the Preferred Reporting Items for Systematic Reviews and Meta‐Analyses extension for Scoping Reviews (PRISMA‐ScR) statement in reporting the utilized methodology, analyses and results (Sarkis‐Onofre et al., 2021; Tricco et al., 2018).

### 3.2 Methods

Two databases for nursing and allied health sciences, CINAHL Complete and Medline, were chosen. The search strategy was tailored with the help of a university's information specialist. After defining and checking the subject headings with MeSH (Medline) and Subject Headings (CINAHL), and after test runs, it was decided to use keyword search. The keyword search was chosen because the subject search yielded a narrower result, and usable and eligible studies were eliminated in trial searches. The search phrases are visible in Table 1.

**TABLE 1:** The search phrases used in CINAHL and Medline databases.

Advanced search in CINAHL and Medline	
AB(^a^ (genomics OR genetics OR genome*)	AND
AB (competenc* OR knowledge OR attitude* OR literacy)	AND
AB (nursing OR nurse* OR midwive* OR midwife*)	AND
TX(^b^ (survey* OR questionnaire* OR measure* OR research OR assessment* OR study OR scale OR studie* OR inventor* OR test*)	

^a^ In the abstract.

^b^ In the text.

Eligibility criteria were discussed and decided among the authors. Research articles focused on the genomic competence of nursing staff were considered the most important. Articles that included nurses as informants among other health professionals were accepted. Both genetic and genomic competence studies were accepted to the review. Research articles on the impact of education were included if the authors had performed a pre‐test on the participants before an education intervention. The pre‐tests demonstrated the basic level of the genomic competence of qualified nurses. Articles related to undergraduate nursing students were not accepted as working nurses were considered to have more experience with the ethical dimensions of genomic knowledge in patient encounters than nursing students.

As this review emphasized general and comprehensive competence in genomics and genetics (G/G), the articles focused on competence of a specific disease or pharmacogenetics were excluded. Peer‐review articles were included if they were published in English in the period 2010–2022. Qualitative, quantitative, and mixed‐method studies were included. With this acceptance of different research methods, the authors strive to achieve an in‐depth understanding of key concepts and to find out the accuracy of the instruments to measure broad and complex competence in genomics. Inclusion and exclusion criteria are presented in Table 2.

**TABLE 2:** Eligibility criteria for the review.

Inclusion criteria	Exclusion criteria
Peer‐review article	Other than research article
English	Language other than English
Published in the period 1/2010–1/2022	Published before 2010
Genomic /genetic competence of nursing staff (RN, APRN, MSN, public health nurses, midwives)	Competence of nursing faculty in educational organization, or a client or population perspective, or other professionals than nurses
Information about the competence of pretested postgraduate nursing students before completing training in genomics	Competence of undergraduate nursing students
Extensive genomic competence measurements	Competence surveyed from one specific or narrow part of genomics/genetics, for example pharmacogenetics, BRCA, sick cell crisis, colorectal cancer, thalassemia
Instrument or questions or accurate competence themes were available	Articles for which instruments or question templates or themes were not available

**FIGURE 1:**
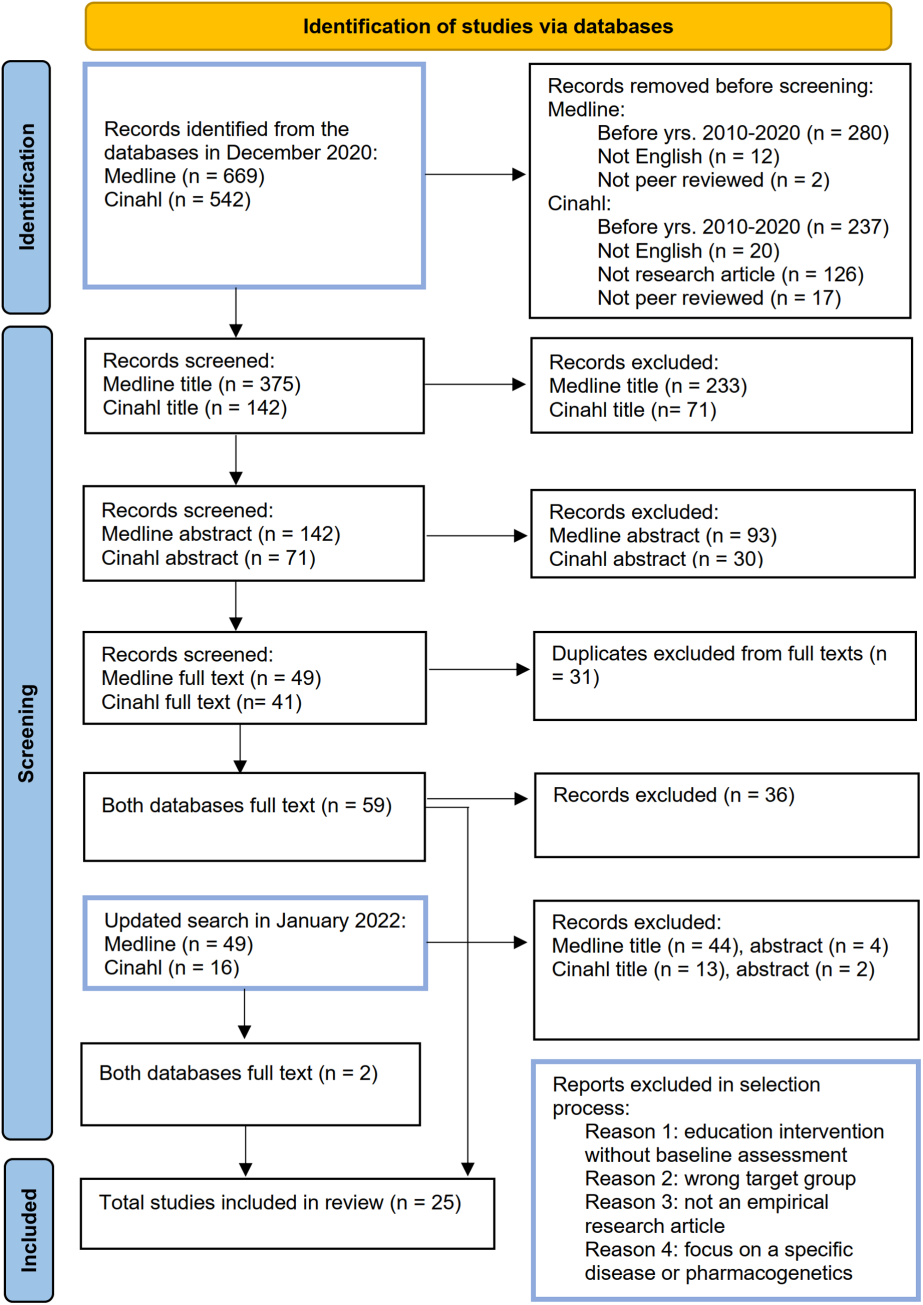
The selection process of eligible articles.

The first author of this article conducted the first phase of the search process in December 2020 and sent the search results (517 articles) to the second author after limiting the search results to peer‐reviewed articles published in English in the period 2010–2020. The search was updated in January 2022 covering articles published from January 2021 to January 2022 to enhance the results with the newest articles. The selection process is illustrated in detail in Figure 1.

The questions or themes of the instruments were clearly described in 16 articles. In addition to this, nine authors were contacted and asked if it was possible to see the original instruments for the analysis of this scoping review to clarify the themes found in the articles. Four out of nine authors sent the additional sources. Five articles whose authors did not send the full instruments were further processed separately and found to contain sufficient information about the content of instruments to perform an analysis. Hence, they were included in the review. In total, 25 articles were selected for inclusion in the analysis.

To increase consistency, the two authors evaluated the title, abstract and full text fully independently. The authors negotiated the search results together and selected the eligible articles. All included articles were saved to RefWorks to make them available for both authors. The included articles are presented in Table 3.

**TABLE 3:** Selected articles.

	Author, year, journal and article name, and country	Objectives	Study type, setting	Participants	Availability of the Instruments	Results	Quality appraisal (JBI)
1	Almomani et al., 2020. PLoS One, 15(6). The Difference in Knowledge and Concerns between Healthcare Professionals and Patients about Genetic‐Related Issues: A Questionnaire‐Based Study Jordan	To assess and compare the knowledge, factors affecting the knowledge and concerns of HCPs and patients regarding genetic‐related issues	A cross‐sectional study; hospital setting	1000 HCPs (76.9%): nurses (38.8%), physicians (33.9%) and pharmacists (27.3%) and 1448 patients	Full instrument available	**Article:** No ethics mentioned **Instrument**: Hidden ethical themes: stigmatization, privacy and confidentiality, consequences for employment and for obtaining health insurance **Results in survey:** HCPs were more concern than patients about issues related to genetic	JBI cross‐sectional 4/8
2	Calzone et al., 2012. Journal of Nursing Scholarship, 44(4). Survey of Nursing Integration of Genomics into Nursing Practice USA	To assess practising nurse attitudes, practices, receptivity, confidence and competency of integrating genomics into nursing practice	A cross‐sectional study; hospital and cancer institution setting	239 licensed registered nurses	Full instrument available, GGNPS	**Article**: No ethics mentioned **Instrument**: Hidden ethical themes: a potential advantages and disadvantages, reliable information	JBI cross‐sectional 6/8
3	Calzone et al., 2013. Personalized Medicine, 10(7). National Nursing Workforce Survey of Nursing Attitudes, Knowledge and Practice in Genomics USA	To assess nursing attitudes, receptivity, confidence, competency, knowledge and practice in genomics to inform education efforts	A cross‐sectional study; setting was not limited neither to hospital nor primary care	619 registered nurses	Full instrument available, GGNPS	**Article**: No ethics mentioned **Instrument**: Hidden ethical themes: as previous GGNPS	JBI cross‐sectional 7/8
4	Calzone et al., 2014. Journal of Nursing Regulation, 5(1). Introducing a New Competency into Nursing Practice USA	The aim of the baseline assessment was to evaluate institutional nursing workforce attitudes, receptivity, confidence, competency, knowledge, and practices regarding genomics.	Part of a longitudinal study; hospital setting	7798 licensed registered nurses	Full instrument available in GGNPS and 2 GKAI questions described and analysed in the article, (GGNPS + GKAI + RACE)	**Article**: No ethics mentioned **Instrument**: Hidden ethical themes: as previous GGNPS	JBI cross‐sectional 6/8
5	Calzone et al., 2016. Journal of Nursing Measurement, 24(1). Test–Retest Reliability of the Genetics and Genomics in Nursing Practice Survey Instrument USA	To measure the reliability of GGNPS instrument and to revise the survey	Part of longitudinal study as cross‐sectional survey; hospital setting	232 registered nurses	Full instrument available, GGNPS	**Article**: No ethics mentioned **Instrument**: Hidden ethical themes: as previous GGNPS	JBI cross‐sectional 7/8
**6**	Calzone et al. 2018. Nursing Outlook, 66(3). Hospital Nursing Leadership‐Led Interventions Increased Genomic Awareness and Educational Intent in Magnet Settings USA	To assess leadership team interventions to improved RNs' capacity to integrate genomics into practice	a longitudinal study with pre‐ and postinterventions; hospital setting	Registered nurses Intervention (N = 196), Controls (N = 492)	Full instrument available, GGNPS	**Article**: Visible ethics in background section: Ethical challenges of new technology increase integration of genomics into education **Instrument**: Hidden ethical themes: as previous GGNPS	JBI quasi‐experiment 8/9
7	Coleman et al., 2014. Journal of Nursing Scholarship, 46(4). Multi‐Ethnic Minority Nurses' Knowledge and Practice of Genetics and Genomics USA	To determine minority nurses' beliefs, practices, and competency in integrating genetics‐genomics information into practice	A cross‐sectional study; setting was not limited neither to hospital nor primary care	389 registered nurses	Full instrument was not available; a compilation of the five instruments(African American nurses, GKAI, HPBR, RACE, GGNPS)	**Article**: No ethics mentioned **Instrument**: Hidden ethical themes: as previous GGNPS	JBI cross‐sectional 6/8
8	Dagan et al., 2021. Journal of Nursing Scholarship; 53:6, Integrating Genomic Professional Skills Into Nursing Practice: Results From a Large Cohort of Israeli Nurses Israel	To explore the association of genomic knowledge, self‐epistemic authority (SEA), perceived importance of genomics in nursing, and the integration of genomic skills into nursing practice	A cross‐sectional study, hospital setting	423 nurses	Full instrument available	**Article**: No ethics mentioned **Instrument**: Hidden ethical themes: Understanding the issue of confidentiality	JBI cross‐sectional 8/8
9	Gharaibeh et al., 2010. International Nursing Review, 57(4). Nurses' and Midwives' Knowledge and Perceptions of Their Role in Genetic Teaching Jordan	To explore Jordanian nurses' and midwives' knowledge and perceptions of their role in genetic teaching.	A cross‐sectional study; hospital setting	200 registered nurses and midwives	Full instrument available	**Article**: Visible ethics in conclusion section: Health services must be provided and developed within a broad ethical framework **Instrument**: No ethics	JBI cross‐sectional 6/8
10	Godino et al. 2013. Journal of Advanced Nursing, 69(5). Knowledge of Genetics and the Role of the Nurse in Genetic Health Care: A Survey of Italian Nurses Italy	To explore nurses' basic knowledge of genetics, their perceptions of the relevance of genetics and their opinions about the role of the genetic nurse	A cross‐sectional study; setting was not limited neither to hospital nor primary care	385 registered nurses	Full instrument available	**Article**: No ethics mentioned **Instrument**: No ethics **Results:** To open‐ended question was answered about the willingness to receive more education from ethical aspects of genetics	JBI cross‐sectional 6/8
11	Lopes‐Júnior et al., 2017. Nursing & Health Sciences, 19(1). Genetic Education, Knowledge and Experiences Between Nurses and Physicians in Primary Care in Brazil: A Cross‐Sectional Study Brazil	To examine genetics education, knowledge, and genetics‐related experiences among nurses and physicians	A cross‐sectional study; primary care settings	54 respondents: 30 nurses and 24 physicians	Full instrument was not available	**Article**: No ethics mentioned **Instrument**: Visible ethical themes: awareness of ethical aspects of genetic counselling Result: 88.9% of respondents were unfamiliar with the ethical aspects of genetic counselling	JBI cross‐sectional 5/8
12	McCabe et al., 2016. Journal of Continuing Education in Nursing, 47(4). Web‐Based Assessment of Genomic Knowledge Among Practising Nurses: A Validation Study USA	To explore the feasibility of a Web‐based version of the GNCI and to test its psychometric performance	Validity evaluation hospital setting	75 registered pediatric nurses	full instrument available The Genomic Nursing Concept Inventory (GNCI)	**Article**: No ethics mentioned **Instrument**: No ethics	JBI cross‐sectional 6/8
13	Melo et al., 2015. Journal of Community Genetics, 6. Genetics in Primary Health Care and the National Policy on Comprehensive Care for People with Rare Diseases in Brazil: Opportunities and Challenges for Professional Education BRAZIL	To analyse genetic competencies of primary healthcare professionals in Brazil.	A cross‐sectional study; primary healthcare setting	45 health practitioners: 21 doctors, 16 nurses, and 8 dentists	Full instrument available	**Article**: Visible ethics in introduction section: ELSI related to genetic testing and genetic data mentioned as part of the core competencies in genetics guidelines Visible ethics in discussion section: training content of genetic counsellors includes ethics **Instrument**: No ethics	JBI cross‐sectional 4/8
14	Murakami et al., 2020. Nursing & Health Sciences. 22 (2). Developing Competencies in Genetics Nursing: Education Intervention for Perinatal and Pediatric Nurses Japan	To develop a genetics nursing seminar, to evaluate learners' awareness of genetics knowledge and confidence in providing nursing care	Prospective pilot study; university setting	15 nurses, 2 midwives and 27 students	Full instrument was not available	**Article**: Ethics in background section, design, and implementation of the education **Instrument**: No ethics in pre‐education assessment; posteducation assessment included several aspects of ethics (not included in this scoping review)	JBI cross‐sectional 7/8
15	Newcomb et al., 2019. Nursing 2019. 49(7). Are Genetics/Genomics Competencies Essential for All Clinical Nurses? USA	To describe the current utilization of genetics/genomics nursing competencies in acute care and to determine whether they perceive the competencies as relevant	A cross‐sectional study; hospital setting	533 registered nurses	Full instrument available	**Article**: no ethics mentioned **Instrument**: Visible and hidden ethics in questions in identifying ethical issues, in decision‐making process, and in clients' rights	JBI cross‐sectional 4/8
16	Plavskin et al., 2019. Nursing open, 6(4). Validity Evaluation of The Genetics and Genomics in Nursing Practice Survey USA	To psychometrically test the Genetics and Genomics Nursing Practice Survey (GGNPS) for evidence of content, face and construct validity.	Validity evaluation, a part of a longitudinal study; hospital setting	6861 registered nurses	Full instrument available, GGNPS	**Article**: No ethics mentioned **Instrument**: Hidden ethical themes: as previous GGNPS	JBI cross‐sectional 6/8
17	Saligan & Rivera, 2014. The Philippine Journal of Nursing, 84(2). Filipino‐American Nurses' Knowledge, Perceptions, Beliefs and Practice of Genetics and Genomics USA	To explore the knowledge, perceptions, beliefs, practice and genomic education of Filipino‐American nurses	A cross‐sectional study; setting was not limited neither to hospital nor primary care	112 Filipino‐American nurses	Full instrument available (compilation of five instruments)	**Article**: No ethics mentioned **Instrument**: Hidden ethical themes: A part of instrument (RACE) is entirely from ethical view: ethnicity. Hidden theme in a question: “Do you believe that genetic testing can be used to discriminate against ethnic minorities?”	JBI cross‐sectional 5/8
18	Santelli, 2016. Journal of Nursing Measurement, 2(4). Development and Psychometric Testing of the Criterion‐Referenced Measurement Tool for Genetics USA	To develop a criterion‐referenced instrument to provide effective documentation of knowledge of advanced practice nursing in genetics	Validity evaluation; setting was not limited neither to hospital nor primary care	356 advanced practice nurses and registered nurses	Full instrument was not available, CRMTG	**Article**: No ethics mentioned **Instrument**: No ethics	JBI cross‐sectional 7/8
**19**	Seven et al., 2015. Nurse Education Today, 35(3). Nurses' Knowledge and Educational Needs Regarding Genetics Turkey	To determine Turkish registered nurses' current knowledge and educational needs in relation to genetics	A cross‐sectional study; hospital setting	175 registered nurses	Full instrument available	**Article**: Visible ethics in background section: nurses do not have adequate knowledge, for example ethical issues **Instrument**: No ethics	JBI cross‐sectional 6/8
20	Wallen et al., 2011. Nurse Education Today, 31. Evaluating a Hybrid Web‐Based Basic Genetics Course for Health Professionals USA	To determine learner outcomes including change in knowledge and self‐efficacy and to explore learner perceptions of the effectiveness of a basic genetics course	A prospective pre–posttest study; hospital setting	129 healthcare providers: nurses involved in clinical research (80), APN (25), nurse manager (7), allied health professionals (14)	Full instrument available	**Article**: Visible ethics in background section: Nurses' roles expansion since science development and ethical, legal, and social implications; education modules including ethics **Instrument**: Hidden ethical themes: employment discrimination, privacy **Results**: pre‐test showed low knowledge in ELSI of genetic testing in minors (15.7% correct) and workplace discrimination (9.4% correct)	JBI cross‐sectional 5/8
21	Whitt et al., 2016. Journal of the American Association of Nurse Practitioners, 28(3). Improving Nurse Practitioners' Competence with Genetics: Effectiveness of an Online Course USA	To assess the effectiveness of an online genetics course for improving nurse practitioners' knowledge, competence, and comfort with genetic principles and their application to clinical practice	A pre–posttest study, Graduate nurse practitioner students, university setting	140 students minimum of a bachelor's degree	Full instrument available	**Article**: Visible ethics in background subdivision: core competencies including ethics, course objectives; in limitation subdivision: instrument did not evaluate ethical issues **Instrument**: No ethics	JBI cross‐sectional 4/8
22	Williams & Dale. 2016. Journal of Nursing Education, 55(10). A Partnership Approach to Genetic and Genomic Graduate Nursing Curriculum: Report of a New Course's Impact on Student Confidence USA	To develop and assess the online course based on the Essential Genetic and Genomic Competencies for Nurses(ANA/ISONG)	A pre–poststudy; university setting	Pre‐course assessment (Graduate nursing students 10 (91%) of 11 students)	Full instrument was not available	**Article**: Visible ethics in background subdivision**:** Course content included ethics **Instrument**: Visible ethics in a competence category question: Confidence in ethical, legal, and social implications **Results:** Very low self‐reported *confidence to own* effective strategies to resolve ethical, legal, and social implications issues related to genetics‐genomics (1.3 + − 0.3) in 1–5 scale.	JBI cross‐sectional 4/8
23	Wright et al., 2019. Journal of Nursing Scholarship, 51(1). Genomic Literacy of Registered Nurses and Midwives in Australia: A Cross‐Sectional Survey Australia	To measure the genomic literacy of Australian registered nurses and midwives	A cross‐sectional study, setting was not limited neither to hospital nor primary care	253 registered nurses (85.7%), and registered midwives (14.3%)	Full instrument available CNCI	**Article**: No ethics mentioned **Instrument**: No ethics	JBI cross‐sectional 6/8
24	Wright et al., 2020. Collegian, 27. Genomics in Oncology Nursing Practice in Australia Australia	To understand how genomics is understood and applied in oncology nursing practice in Australia	Qualitative Semi‐structured interviews; hospital setting	9 registered oncology nurses	Question themes available	**Article**: No ethics mentioned **Instrument**: No ethics	JBI qualitative 9/10
25	Yeşilçinar et al., 2022. Genetics and genomic competency of Turkish nurses: A descriptive cross‐sectional study Turkey	to assess the genetic and genomic competency of Turkish nurses in practice.	A descriptive cross‐sectional research; clinical or academic setting	385 nurses	Full instrument available, GGNPS	Article: No ethics mentioned Instrument: Hidden ethical themes: as previous GGNPS	JBI cross‐sectional 6/8

Although the main interest of this scoping review was not the findings of the studies, but rather the content concerning ethical issues of instruments, the critical appraisal tools of the Joanna Briggs Institute (JBI, n.d. were independently used by the first two authors in assessing the articles. The Quasi‐Experimental tool was used in Calzone, Jenkins, et al. (2018) article. Wright et al.'s (2020) semi‐structured interview article was rated with the Qualitative tool. The Checklist for Analytical Cross‐Sectional Studies was utilized in other articles.

The scores of the appraisal differed between the two authors in 17 articles. The differences mostly concerned the objective, standard criteria used for the measurement of the condition, or the strategies used to deal with stated confounding factors. If there was only a one‐point difference, the authors selected the lower score. If there was a larger gap in the scores, the authors explored the study again, deliberated and reached a conclusion for the quality assessment. The results of the quality appraisal are presented in Table 3.

### 3.3 Analysis

After sources had been selected for inclusion, data were extracted from each article. Two data extraction sheets were developed for collecting information. The articles were examined from the perspective of the research question, and discovered themes were extracted and categorized. The first formatted sheet included authors, year of publication, journal, the title of article, objectives, study type and setting, participants, availability of the instrument, results and quality appraisal. This data sheet is seen in Table 3. Another sheet was utilized in the writing process to gather information including the concepts, instrument details, measurement details, field of genomics / genetics and the content of the competence instruments. The synthesis process was started by piloting the forms with three articles (Pollock et al., 2021).

The thematic analysis method used in the review was inductive with the focus on ethics. Both a semantic and latent approach were used to make all relevant data extracts related to ethics in genomics visible (Vaismoradi et al., 2013). This enabled the authors to visualize main content and underlying assumptions concerning the data. In the synthesis process, the concepts and themes found in the instruments or articles were discussed to form a joint understanding of them. The identified themes of ethical competence in genomics in nursing are described in the results.

### 3.4 Ethics

Research Ethics Committee approval was not required in scoping review.

## 4 RESULTS

### 4.1 Characteristics of the included studies and instruments

In this study, 25 articles were analysed. The selected studies were conducted in USA (n = 14), Australia (n = 2), Brazil (n = 2), Jordan (n = 2), Turkey (n = 2), Israel (n = 1), Italy (n = 1) and Japan (n = 1). In total, 19 different instruments were identified in these 25 articles. One instrument, The Genetics and Genomics in Nursing Practice Survey (GGNPS), was utilized in six articles (Calzone et al., 2012, 2013, 2016; Calzone, Jenkins, et al., 2018; Plavskin et al., 2019; Yeşilçinar et al., 2022) and one instrument, the Genomic Nursing Concept Inventory (GNCI), in two articles (McCabe et al., 2016; Wright et al., 2019). In addition to this, GGNPS was utilized as the basis of two compilation instruments (Calzone et al., 2014; Coleman et al., 2014).

The primary concepts were genomic competence and nurses' ethical competence in genomics which were explored in this review. The concepts of genetics and genomics were used in parallel, alternately and separately throughout the articles and instruments. Only few of the studies (Dagan et al., 2021; Murakami et al., 2020; Newcomb et al., 2019; Saligan & Rivera, 2014; Wright et al., 2019) provided definitions for the concepts. Genetics referred to information about individual genes and their impact on single‐gene disorders. Genomics was described as a study of all the genes including gene–gene and gene–environment interactions, and the influence of psychosocial and cultural factors. The scoping showed that every instrument included genetic questions, none of the instruments included only genomic questions, and 15 of 25 instruments included questions categorized to both genomic and genetic areas.

The concept of competence was used alongside with knowledge, literacy (Wright et al., 2019), clinical performance as skills (Calzone et al., 2013) and attitudes (Calzone et al., 2012). Calzone et al. (2013) described that competences are well‐established, which might reveal why competences were not defined more in the articles. Most of the articles referred to the Essential Nursing Competencies and Curricula Guidelines for Genetics (Consensus Panel, 2009) as a basis of competence. There was a consensus among the authors that basic genetic/genomic competences are expected from all nurses regardless of their level of academic education (e.g. Calzone et al., 2013; Coleman et al., 2014; Melo et al., 2015; Newcomb et al., 2019; Yeşilçinar et al., 2022).

The instruments in the articles were developed based on literature reviews, the authors' expertise, collaboration with specialists, previous instruments or previously published competence guidelines by global and national nurses' associations, and education organizations such as the International Society of Nurses in Genetics, Consensus Panel, American Nurses Association and the National Coalition for Health Professional Education in Genetics.

Competence instruments included the assessment of knowledge, skills, attitudes, confidence, beliefs, perceptions or concerns. They measured actual or self‐reported competence. An actual competence section was found in 19 articles, while ten articles included also self‐estimated competence. Five articles included only self‐evaluation of competence. Santelli (2016) article did not report this information. The majority of the studies were quantitative, only Wright et al. (2020) study was constructed for qualitative methods with semi‐structured questions.

### 4.2 Ethical competence as an unstructured phenomenon in genomics

Ethics was the primary focus in this review. The description of ethics was unstructured and scattered in the articles. The articles or instruments included visible or hidden ethics.

In seven articles, six different instruments did not include any visible or hidden ethical questions (Gharaibeh et al., 2010; Godino et al., 2013b; McCabe et al., 2016; Melo et al., 2015; Seven et al., 2015; Whitt et al., 2016; Wright et al., 2019). These instruments were fully available for analysis. In addition, three instruments which were not fully available did not cover topics of ethics either (Murakami et al., 2020; Santelli, 2016; Wright et al., 2020).

### 4.3 Visible ethics

Of the 25 articles, only 11 (Calzone, Jenkins, et al., 2018; Gharaibeh et al., 2010; Godino et al., 2013b; Lopes‐Júnior et al., 2017; Melo et al., 2015; Murakami et al., 2020; Newcomb et al., 2019; Seven et al., 2015; Wallen et al., 2011; Whitt et al., 2016; Williams & Dale, 2016) mentioned the word ethics (ethic, ethical) in the article or in the instrument. These were categorized as visible ethics. Only three instruments asked questions that included the word ethics (ethic, ethical) (Lopes‐Júnior et al., 2017; Newcomb et al., 2019; Williams & Dale, 2016). The references to ethical approval of the study process were excluded from this inspection.

In the category of visible ethics, Murakami et al. (2020) education intervention study perceived ethics as a vital aspect of genomics, and this was mentioned several times in the article. However, the assessment of self‐reported pre‐education genetics knowledge in the study did not include ethical questions. In the posteducation assessment, which was not analysed in this review, ethics was included in the qualitative part of the study (Murakami et al., 2020). The same phenomenon was visible in Whitt et al. (2016) study. They reported that the course objectives included ethical issues. However, the instrument did not evaluate competences related to ethics. Wallen et al. (2011) described ethical and social challenges as one of the contents of their education module. In contrast with Williams and Dale (2016) study, which managed to increase knowledge in ethics, Wallen et al. (2011) described continuing gaps in ethical knowledge after the education. Visible ethics in education were described as challenges related to the curriculum process, course objectives, a lack of teaching material that included ethical approach and a desire to learn more in continuous learning.

Three instruments directed ethical questions to nurses in relation to their skills and knowledge. Confidentiality in resolving ethical problems, familiarity with ethical aspects of genetic counselling and ability to identify ethical issues were asked about. In a study of Williams & Dale (2015), the nurses' self‐reported confidence was low in the area of using strategies in resolving ethical, legal and social implication issues related to genetics‐genomics (1.8 + − 0.2) on a scale of 1–5. In an article concerning the primary healthcare setting, 88.9% of professionals (nurses and physicians) were unfamiliar with the ethical aspects of genetic counselling (Lopes‐Júnior et al., 2017). Newcomb et al.'s (2019) instrument asked nurses about their ability to identify ethical, cultural, legal, ethnic, religious, fiscal and societal issues as a part of genomics in a single question. The results of the question were not presented in their article. The nurses' ability to identify ethical issues appeared on three levels in the articles: identifying a knowledge gap in themselves (Godino et al., 2013b; Wallen et al., 2011), ethical issues in practice (Newcomb et al., 2019; Williams & Dale, 2015) and development needs in the healthcare community (Gharaibeh et al., 2010).

### 4.4 Hidden ethics

In thirteen articles, the instruments considered ethics‐related themes were categorized as hidden ethics (Calzone et al., 2012, 2013, 2014, 2016; Calzone, Jenkins, et al., 2018; Coleman et al., 2014; Dagan et al., 2021; Melo et al., 2015; Newcomb et al., 2019; Plavskin et al., 2019; Saligan & Rivera, 2014; Wallen et al., 2011; Yeşilçinar et al., 2022). The instrument of Newcomb et al. (2019) included both visible and hidden ethics.

The themes of ethics were addressed as skills, concerns, advantages and disadvantages in the instrument questions of hidden ethics. The skills included nurses' ability to define clients' autonomy, informed decision‐making, voluntary action (Newcomb et al., 2019) and in a case description by Melo et al. (2015), as a nurses' ability to act ethically. Ethical themes in the professionals' concerns included costs, stigmatization, privacy, confidentiality, insurance, employment discrimination (Almomani et al., 2020) and the discrimination of ethnic minorities (Saligan & Rivera, 2014). Workplace discrimination was also addressed in Wallen et al.'s (2011) study.

Eight studies in the category of hidden ethics (Calzone et al., 2012, 2013, 2014, 2016; Calzone, Jenkins, et al., 2018; Coleman, 2014; Plavskin et al., 2019; Yeşilçinar et al., 2021) measured competence using GGNPS instrument or a compilation of several instruments including GGNPS. In GGNPS, the ethical issues were referred to as potential advantages and disadvantages. The advantages addressed treatment decisions and improved services. The disadvantages addressed time‐consuming practices, expenses, anxiety of patients and insurance discrimination. (Calzone et al., 2012, 2013, 2014, 2016; Calzone, Jenkins, et al., 2018; Coleman et al., 2014; Plavskin et al., 2019; Yeşilçinar et al., 2021). In the articles using a compilation of instruments (Calzone et al., 2013, 2014; Coleman et al., 2014), ethics was not addressed outside GGNPS.

### 4.5 Ethics as part of an extensive competence

Overall, ethics was discussed illogically. Ethics was described as part of competence, but it was not necessarily assessed with the instrument. It was typical to the instruments that ethical competence was measured as part of a large set of other issues related to genomics. This set included ethical, legal, social (Melo et al., 2015; Williams & Dale, 2015), religious, cultural and ethnic issues (Newcomb et al., 2019). It was also common to use a question type that condensed several ethical principles and skills into a single competence and did not separate the parts into distinct functions: “I define issues that undermine the rights of my clients for autonomous, informed genetic‐ and genomic‐related decision making and voluntary action.” (Newcomb et al., 2019).

**FIGURE 2:**
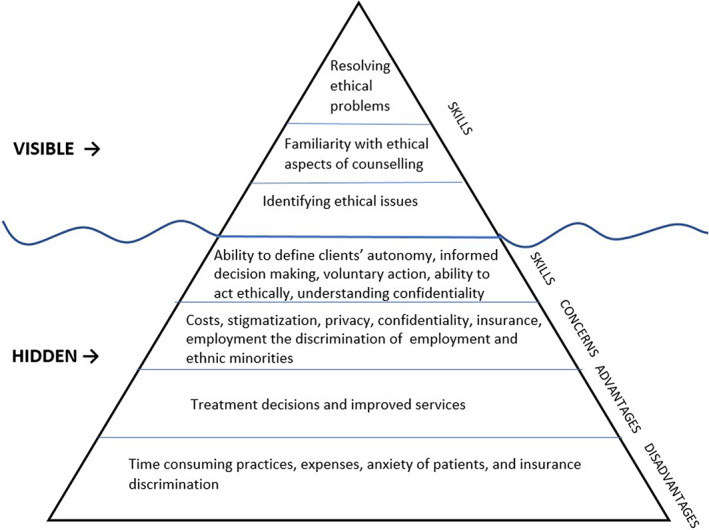
The iceberg‐shaped summary of the visible and hidden ethics in the instruments.

The verbs to resolve, know, argue, provide, define and identify were used to describe practice‐related skills in ethical competence in instruments. When a thematic analysis allows interpretation (Vaismoradi et al., 2013), ethical principles can be found in the categories of visible and hidden ethics. The identified ethical principles included justice, privacy, respect for autonomy, patient rights for optimal care, beneficence, effectiveness and nonmaleficence. The iceberg‐shaped illustration of ethics in the instruments is presented in Figure 2.

## 5 DISCUSSION

It can be seen in the results of this review that ethical perspectives were considered inconsistently in the articles and instruments. If ethics was discussed it was done illogically. It was described as a part of competence, but it was not necessarily assessed with an instrument. Only three articles asked questions that included word ethic (ethics, ethical). Most articles demonstrated ethics as a hidden issue. If ethics is not directly referred to by its name it may be ignored completely, ethical issues may be difficult to identify in practice, or ethics may be perceived as belonging to the role of other professionals. In addition, if ethics is too abstractly or unclearly described in the instruments, it leaves too much room for interpretation for survey respondents. And the answers can be misinterpreted. Nine different instruments did not include visible or hidden ethical questions at all. A previous review by Skirton et al. (2012) had similar findings: only 3 articles out of 11 mentioned ethical concerns.

Many associations, frameworks and guidelines perceive ethics as one of the competences of genomics (American Association of Colleges of Nursing [AACN*]*, 2021; Consensus Panel, 2009), and nurses have considered genomics as an important area in health care (Calzone et al., 2014; Coleman et al., 2014); however, professionals have felt inadequately prepared to implement genomics in their practice (Calzone et al., 2013). The ethical challenges that arise in genomics can cause this lack of confidence for implementation if nurses do not have skills to resolve them. Resolving ethical problems was described as one of the competence areas in which nurses' confidence was the lowest in Williams & Dale's (2015) study. Similarly, in an integrative review, Wright et al. (2017) discussed the connection between a lack of confidence in using genomics in practice and a lack of knowledge. The results indicate that it is important to strengthen ethical basis in education to implement genomics ethically and to develop a sustainable instrument for research.

According to the results, the ethical issues were placed in one extensive area of competence. One instrument question utilized to measure ethical competence could combine all ethical, legal, social, religious, cultural and ethnic issues into a single assessed skill. Ethical issues in genomics deserve their own part in competence instruments. Ethical issues are considered in a different way compared to, for example, legal or cultural issues, although these all have some similarities. The results also demonstrated that ethical skills were combined into a single skill that included different aspects of ethics: the respect of autonomy as a principle, and guiding clients in informed consent process as a skill. In this process, nurses need to have competences for describing the nature of a genetic test, explain how test results will be delivered, explain the limitations and advantages of the test, potential findings and their implications to patients and their family, offer additional information resources in a language the patient understands and explain where data will be stored and who can access it (Tluczek et al., 2019). Voluntariness, confidentiality, privacy and the right not to know are additional principles in the informed consent process besides the respect for autonomy which was presented in the question.

These dimensions of ethics have been bound together in the instruments of this review similarly as they have been described in the competence recommendations for genomics in nursing (Consensus panel, 2009; Greco & Salveson, 2008). The guidelines for identifying ethical, ethnic, cultural, legal, fiscal and social issues (Consensus panel, 2009) give a false idea that only one skill is enough for performing all these issues. The description of one skill is reflected up to the guidelines‐based instruments and even to practice level.

The general ethical competence of nurses has been surveyed from the seven viewpoints: ethical decision‐making, reasoning, sensitivity, reflection, knowledge, behavior and action (Poikkeus et al., 2014). This wide spectrum of ethics and direct questions about ethical competences are vital to be included in design of future instruments. Only decision‐making, knowledge and action were aspects included in some of the identified instruments of this scoping review.

This review contributes to nursing science and nursing education by increasing understanding of how comprehensive genomic competence is. Ethics, as part of genomic competence, should be taught and researched in more detail. The role of nurses will be emphasized in the future as genomic information and knowledge of patients increase further.

### 5.1 Limitations

The use of only two databases for the literature search may be considered as a limitation for this review. However, the search was made with an information specialist of health sciences and search phrases were formulated after comprehensive consideration and pilot searches. The pilot searches illustrated that the chosen databases were extensive, and they systematically and worldwide yielded information about the instruments used for assessing genomic competence in nursing. Used databases covered the research area of nursing competence and genomics in nursing.

The well‐considered decision about choosing these databases was based on the following evidence. Subirana et al. (2005) stated that for a search for a systematic review on nursing topics, CINAHL and MEDLINE are essential databases for accuracy of the search. CINAHL is considered the world's largest source of full‐text nursing and allied health journals, and it is an essential database to clinical practice and research in nursing. (Ebsco, n.d.; Hopia & Heikkilä, 2020; McGill Library, n.d.). Medline database is also widely recognized as an important source for biomedical literature (Tampere University Library, n.d.).

Due to limited access to full instruments in some articles, assessing each question in detail was not possible. However, the content of the questions was available. In addition, all articles included described the competence questions sufficiently extensively even if the full instruments were not available in additional sources or the authors did not provide their original instruments for the purposes of this review.

### 5.2 Conclusion

Ethical aspects are recommended to be included in education and clinical practice of nursing. In addition, it is stated to be part of genomics. Thus, competence instruments should also systematically include ethics to measure this area of genomics. Current competence instruments measure ethics only as part of a large entity together with other issues such as legal, social, cultural and ethnic issues. It is important to refine ethical aspects of genomic competence in nursing practice and to measure perceptions, skills, justifications and concerns related to ethical competence in genomics with further developed instruments.

## AUTHOR CONTRIBUTION

All authors have made substantial contributions to conception and design. M.L implemented the first phase of the search. M.L and E.A implemented the selection and quality appraisal process. M.L, A.H and E.P took part to the analysis of the results M.L wrote the manuscript with support from E.A, A.H and E.P. All authors made critical revision of the article and have approved the final version to be published.

## FUNDING INFORMATION

This research received no specific grant from any funding agency in the public, commercial or not‐for‐profit sectors.

## CONFLICT OF INTEREST STATEMENT

No conflict of interest has been declared by the authors.

## Data Availability

The data that support the findings of this study are available on request from the corresponding author. The data are not publicly available due to privacy restrictions. Part of the instruments analyzed were available only for this review.
